# Effects of graphene intercalation on dielectric reliability of HfO_2_ and modulation of effective work function for Ni/Gr/c-HfO_2_ interfaces: first-principles study

**DOI:** 10.1038/s41598-018-19411-0

**Published:** 2018-01-18

**Authors:** Kehua Zhong, Yanmin Yang, Jian-Min Zhang, Guigui Xu, Zhigao Huang

**Affiliations:** 1 Fujian Provincial Key Laboratory of Quantum Manipulation and New Energy Materials, College of Physics and Energy, Fujian Normal University, Fuzhou, 350117 People’s Republic of China; 2 Concord University College, Fujian Normal University, Fuzhou, 350117 China; 3 Fujian Provincial Collaborative Innovation Center for Optoelectronic Semiconductors and Efficient Devices, Xiamen, 361005 China

## Abstract

We have investigated the effects of graphene intercalation on dielectric reliability of HfO_2_ for Ni/Gr/HfO_2_ interfaces, and the effects of graphene intercalation and interfacial atom vacancy on the effective work function (EWF) of Ni/Gr/HfO_2_ interfaces using first-principle calculation based on density functional theory. The calculated results indicate that graphene intercalation can improve dielectric reliability of HfO_2_ dielectric even for the interfaces having interfacial oxygen vacancy or a small amount carbon vacancy. Moreover, the calculated results indicate that, inserting graphene into Ni/HfO_2_ interface induces the EWF’s to decline, and controlling interfacial oxygen or carbon vacancy can effectively tune the EWF of Ni/Gr/HfO_2_ interface. Our work strongly suggests that the use of graphene synthesized into Ni/HfO_2_ interface is a very effective way to improve the interface quality, and controlling interfacial oxygen or carbon vacancy is also an attractive and promising way for modulating the EWF of Ni/Gr/HfO_2_ interfaces.

## Introduction

Metal gate and high dielectric constant “high-*k*” gate dielectric are considered essential in continued downscaling of metal-oxide-semiconductor field-effect transistor (MOSFET)^1,2^. Having appropriate effective work function (EWF) and good dielectric reliability are crucial requirements for desired metal gate and gate dielectric, respectively. However, during the actual sample deposition and integration procedures, metal gate inevitably reacts with gate dielectric substrate. This reaction between them can induce metal contamination in thin gate dielectric^3–5^ and fermi level pinning^6^ which makes the EWF of gate electrode to deviate from the desired value. As a result, reliability and performance of device has been degraded. Therefore, improving dielectric properties of thin gate dielectric and effective tuning work function are essential for reliable integration of MOS devices. Yet, as well known, proper tuning the EWF is still difficult because of its extreme sensitivity to many interfacial factors^7,8^. It has been reported that processing conditions of device may strongly affect the EWF^9,10^. And then in the actual experimental process, there may exist many defects in the interface of metal/oxide structure. For example, with the help of the oxidation of underlying silicon, oxygen vacancies in HfO_2_ can be easily generated^11,12^. These interface defects would alter the interface structure and then interface dipole. As a result, the EWF is changed.

Replacement gate process which avoids high temperature processing after metal gate deposition could address some concerns induced by the reaction between metal gate and gate dielectric substrate^13,14^. But, it is too complex to be commonly used. Because of graphene’s two-dimensional sheet with monoatomic thickness, the exceptionally high conductivity and high thermal stability, it has a great potential for application in both low-dimensional sciences and nano-scale electronic devices. For example, it has been used as a gate stack electrode in MOS technology^15,16^. Recently, the experimental research^17^ has shown that the use of graphene (Gr) as a gate electrode instead of metal on a high-k gate dielectric improves the gate dielectric quality. Furthermore, several experimental studies on the work function tuning of metal-graphene stack electrode also have been done for metal-graphene-oxide structure^15,16,18^. Among them, Misra *et al.*^18^ explored multilayer graphene as metal gate electrode by inserting it between SiO_2_ dielectric and TiN metal. They found that incorporation of graphene between SiO_2_ dielectric and TiN metal gave rise to significantly improved dielectric reliability and an EWF tuning of gate electrode up to 0.5 eV by controlling the number of graphene layers. And Song *et al*. found the work function for graphene-metal electrode varied depending on the metal species, and it was either pinned to the work function of metal or pinned to a particular value regardless of the work function of metal^16^. They also found that varying the number of graphene layers can tune the work function of graphene/metal from 4.3 eV to 5.1 eV^15^. It has been experimentally investigated that the effects of incorporation of graphene on gate dielectric reliability and work function tuning for metal-oxide interface. Unfortunately, so far, there is little theoretical research on this subject. This motivates us to investigate the impacts of intercalation of graphene into metal/oxide structure.

Currently, since hafnium oxide (HfO_2_) has high dielectric constant and excellent thermal stability and satisfies various technical requirements etc, it has emerged as one of the most preferred gate oxide in metal/high-*k* oxide^2,7^. Furthermore, due to its high work function, thermal stability and good compatibility with high-k oxide, Ni has become a more ideal gate metal material. In this paper, we used first-principles calculations based on density functional theory (DFT) to investigate the following two aspects: (1) the effects of graphene intercalation on dielectric reliability of HfO_2_. (2) the effects of graphene intercalation and interfacial oxygen or carbon vacancy on the EWF of Ni/Gr/HfO_2_ interface.

## Computational Details

**Figure 1:**
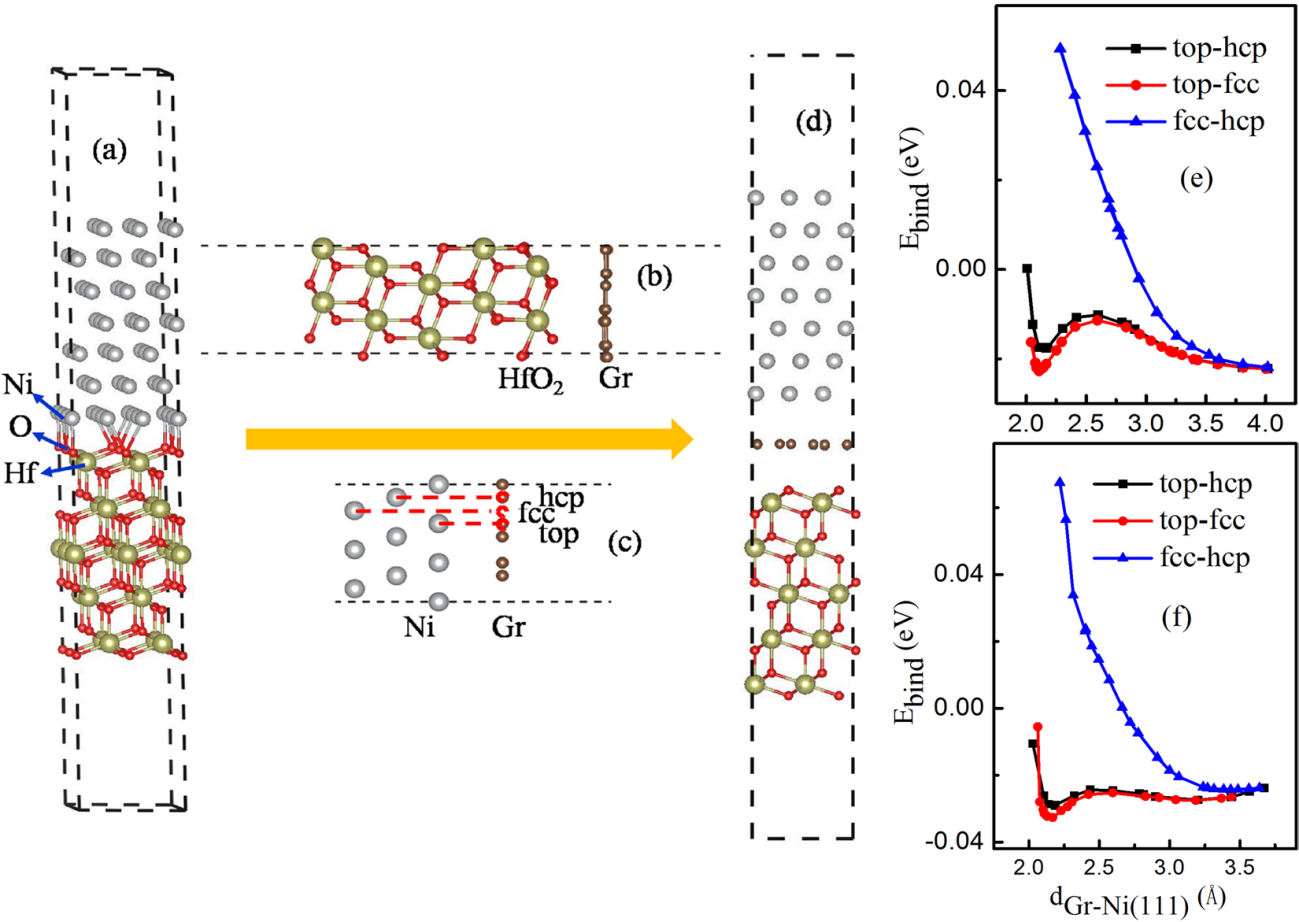
Crystal structure illustrations of supercell for (**a**) Ni(111)/HfO_2_(111), (**b**) Gr/HfO_2_(111), (**c**) Gr/Ni(111), and (**d**) Ni(111)/Gr/HfO_2_(111) interfaces. Binding energy of Gr/Ni(111) surfaces with graphene adsorption in top-fcc, top-hcp and fcc-hcp configurations vs distance *d*_Gr-Ni(111)_ calculated with (**e**) PBE, (**f**) optB88-vdW functional. *d*_Gr-Ni(111)_ is the distance between Gr and Ni(111).

All calculations were carried out using Vienna ab initio simulation package (VASP) with projector augmented wave approach^19–25^. For Ni/HfO_2_ interface, the exchange correlation energy was calculated using PBE generalized gradient approximation (GGA). Since the non-local dispersive interaction of graphene with metal Ni surface is very important and must be considered according to previous researchers^26–28^, then in our concerned Gr/Ni, Gr/HfO_2_ and Ni/Gr/HfO_2_ interfaces without or with atom vacancy, van der Waals contributions should also be considered. And here we employed optB88 exchange functional to describe van der Waals forces. The more details are presented in the back section. Spin polarization was included, and the plane-wave basis cutoff was set at 400 eV which shows a good convergence. A dipole correction was applied to avoid spurious interactions between periodic images of the slab. A 4 × 4 × 1 Monkhorst-Pack k-mesh was adopted for the calculations. The atoms were fully relaxed through the conjugate-gradient algorithm until the residual force on each atom was less than 0.03 eV/Å. Our calculated lattice constants for bulk cubic-HfO_2_ and fcc-Ni are 5.03 Å and 3.51 Å, respectively, which are slightly lower than the experimental values of 5.08 Å^29^ and 3.52 Å^30^. Ni(111) and HfO_2_(111) surfaces were chosen as the building blocks to build Ni/HfO_2_ interface, because both of them were the close-packed and most stable facets^31^. The 3 × 3 Ni(111) primitive surface unit cell has very good lattice match to 2 × 2 HfO_2_(111) primitive surface unit cell with a lattice mismatch about 5%. The Ni(111)/HfO_2_(111) structure used to mimic the Ni/HfO_2_ interface is similar to that previously reported by Li *et al*.^32^. The Ni(111)/HfO_2_(111) interface supercell was constructed by stacking 7 Ni layers on HfO_2_(111) slab (5 Hf layers and 10 O layers) and a 15Å-vacuum which is large enough to avoid any spurious interaction between the periodic replica. The supercell of the interface is shown in Fig. 1(a). During structure relaxation, in the interfaces formed by HfO_2_ base considered in this study, the lateral lattice parameters were fixed to be the value of HfO_2_(111) and two Hf bottom layers and four O bottom layers were frozen to act as HfO_2_ bulk. The Ni(111) surface was then fully relaxed. It may correspond to the features of Ni experimentally deposited on HfO_2_ substrate.

## Results and Discussion

### Interface structure of Ni(111)/HfO_2_(111) with graphene intercalation

The stability of interface was examined by calculation of the binding energy ${E}_{bind}$, which was calculated as

1 $${E}_{bind}=\frac{1}{S}({E}_{AB}-{E}_{A}^{slab}-{E}_{B}^{slab}).$$

Where ${E}_{AB}$, ${E}_{A}^{slab}$, ${E}_{B}^{slab}$ represent the total energies of the A/B interface system, the isolated A and B slabs, respectively. *S* is the cross-sectional area of supercell. With this definition, a negative ${E}_{bind}$ indicates that interface should be stable. Our calculated value of binding energy for Ni(111)/HfO_2_(111) interface was −0.027 eV/Å^2^. It implies that metal Ni(111) surface would energetically favorable to combined with HfO_2_(111) to form Ni(111)/HfO_2_(111) interface.

To study the effects of graphene intercalation on dielectric properties of HfO_2_ and the EWF of Ni/HfO_2_ interface, we need to obtain a stable structure for Ni(111)/Gr/HfO_2_(111) interface. In order to get the stable Ni(111)/Gr/HfO_2_(111) interface structure, firstly, we built interface structure of graphene absorbed on HfO_2_(111) and then found the appropriate structure for Gr/HfO_2_(111) system. Generally, there are three different terminations: O-term, Hf-term and OO-term (terminated by two O layers) for HfO_2_(111) surface. According to refs^33,34^, the O-term surface was found to be energetically stable under O-rich condition. Therefore, to simplify, the graphene was considered to combine onto O-term surface of HfO_2_. Gr/HfO_2_(111) was modeled by a slab model that (3 × 3) graphene was deposited on (2 × 2) HfO_2_(111) surface with five Hf-O trilayers, since they had very good lattice match with a lattice mismatch about 4%. And a 15 Å-vacuum region was also inserted along the direction perpendicular to graphene. In view of previous studies^35,36^, we considered four binding configurations of graphene on HfO_2_(111) substrate. In these configurations, a C atom is adsorbed directly above the center of a Hf-O bond (Bridge), above the hollow site at the center of the Hf-O hexagonal ring (Hollow), on the top of a Hf atom (top-Hf), and on the top of an O atom (top-O), meanwhile other C atoms are adsorbed above HfO_2_(111) surface according to the structure of graphene. The binding energies of Gr/HfO_2_(111) systems for all binding configurations were calculated according to Eq. (1), as shown in Table 1. The binding energies are all negative and differ little. Negative binding energies suggest that graphene is easily deposited on HfO_2_(111) substrate. More, we find that graphene is adsorbed on the HfO_2_ substrate with the average separation between graphene and topmost O layer about 2.91–2.96 Å which is very close to the reported values of 3.05 Å in ref.^35^ and 3.03. Å in ref.^36^. For simplicity, in subsequent study for Ni(111)/Gr/HfO_2_(111) interface, we used the beforehand generated Gr/HfO_2_(111) structure with top-O configuration to build Ni(111)/Gr/HfO_2_(111) interface structure further.

**Table 1:** Calculated results of binding energy and the average separation between graphene and topmost O layer.

	Binding energy (eV/Å^2^)	d_Gr-HfO2_(Å)
Bridge	−0.070	2.95
Hollow	−0.070	2.96
Top-Hf	−0.069	2.91
Top-O	−0.071	2.94

The appropriate structure for Gr/HfO_2_(111) system has been acquired. Nextly, interfacial bonding between graphene and Ni(111) was considered. The graphene/metal systems have been extensively studied^26–28,33,37–39^. Several authors have investigated them with standard DFT approaches where the van der Waals (vdW) contributions are neglected^37,38^. Yet as we all know, it fails to reproduce non-local dispersive interaction which is important in weak binding between graphene and metal surface. Recent studies^26,28^ have evidenced that the optB88-vdW functional has been found to be an effective solution to describe vdW contributions, which plays an important role in binding between graphene and metal surface. Therefore, to reasonably consider the interaction between metal slab and graphene, we employed the vdW density functional in form of the optB88-vdW. Moreover, the three typical symmetric positions of Ni(111) surface which are located above Ni atoms in the first layer, the second layer and third layer are named as top, hcp and fcc position^27,28,38,40^, respectively, as shown in Fig. 1(c). And three typical binding configurations were taken into account in the combining of graphene onto Ni(111) surface. Their representative carbon atoms are located on top and fcc positions, on top and hcp positions, fcc and hcp positions, respectively. Figure 1(e) and (f) show our calculated binding energy curves with PBE functional and optB88-vdW functional, respectively. As shown in Fig. 1(e), the calculated result with PBE functional only appears one predictable minima. But from Fig. 1(f), it can be apparently seen that the calculated results using optB88 exchange functional have reproduced quite well two minima for Gr/Ni(111) system. One locates around 2.1 Å, which is a chemical adsorption distance; while another locates around 3.2 Å, which is a typical physical adsorption distance. The calculated results above are consistent with those previously reported^26,28^. Therefore, in subsequent study for Ni(111)/Gr/HfO_2_(111) interfaces, we employed the optB88 exchange functional to describe van der Waals forces. In addition, from Fig. 1(f), it can be clearly found that the binding energies for graphene adsorbed on Ni(111) with fcc-hcp are significantly higher than that with top-fcc and top-hcp. And the binding energies for the systems with top-fcc and top-hcp are almost the same. So, in this work, it is only considered that Ni(111) combines with graphene in forms of top-fcc and top-hcp.

The Ni(111)/Gr/HfO_2_(111) interface was built by combining Ni(111) with the previously acquired Gr/HfO_2_(111) structure. Assume that Ni and graphene being deposited on a prefabricated HfO_2_ substrate to form an interface. So in structure relaxation, the lateral lattice parameters were fixed to be the value of c-HfO_2_(111) and the Ni(111) surface and graphene were then fully relaxed in finding the equilibrium structure of interface. The schematic for the supercell of the Ni(111)/Gr/HfO_2_(111) supercell is shown in Fig. 1(d). Their binding energies are about −0.235 eV/Å^2^ and −0.230 eV/Å^2^ for top-hcp and top-fcc, respectively. Noted that the binding energies for the two binding configurations are all negative and their difference is little. Thus it is reasonable to infer that Ni(111) energetically prefers to combine with Gr/HfO_2_(111). Moreover, it is found that for both binding configurations, Ni(111) slab is bound with Gr/HfO_2_(111) interface in a typical physical adsorption distance. The average separations between graphene and the nearest neighbor Ni layer are about 3.20 Å and 3.27 Å for top-fcc and top-hcp, respectively.

### Effects of graphene intercalation on effective work function for Ni(111)/HfO_2_(111)

**Figure 2:**
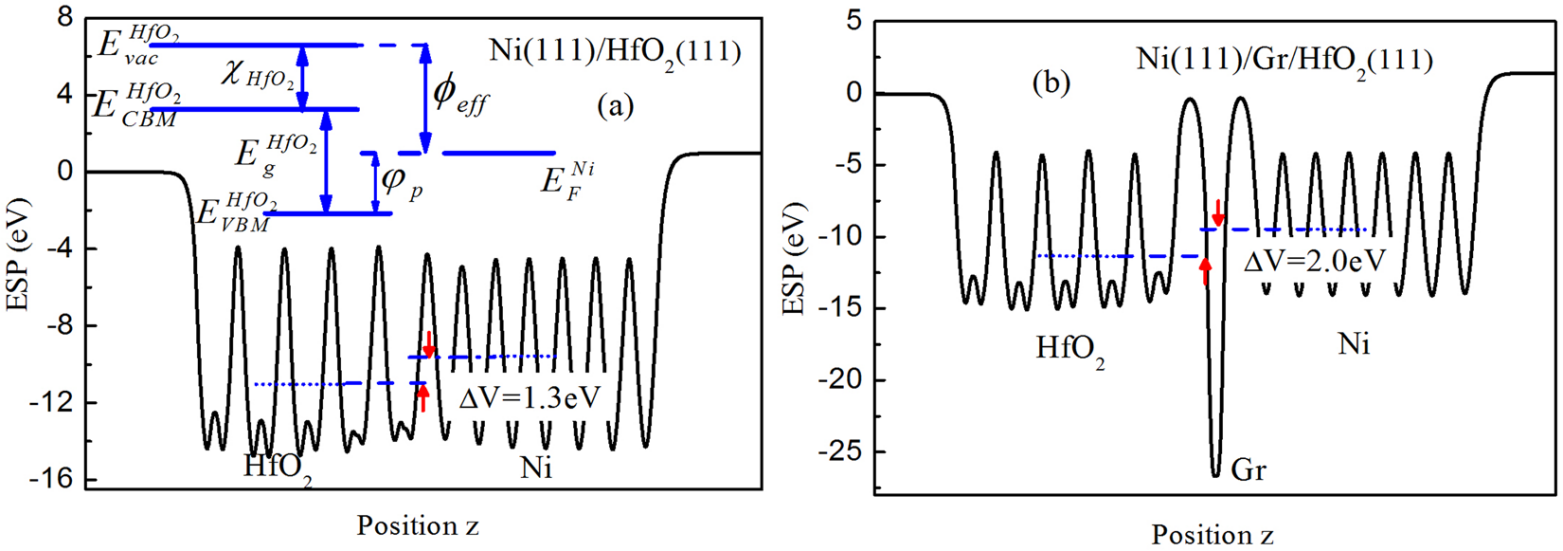
Plane-averaged potentials for (**a**) Ni(111)/HfO_2_(111) interface and (**b**) Ni(111)/Gr/HfO_2_(111) interface.

The interface EWF can be estimated using potential-line-up method, as schematically shown in Fig. 2(a), and the interface EWF ${\varphi }_{eff}$ is generally estimated as follows^41–43^

2 $${\varphi }_{eff}=({\chi }_{Hf{O}_{2}}+{E}_{g}^{Hf{O}_{2}}+{E}_{VBM}^{Hf{O}_{2}}-{E}_{F}^{Ni})-\Delta V.$$

Where ${\chi }_{Hf{O}_{2}}$, ${E}_{g}^{Hf{O}_{2}}\,$and ${E}_{VBM}^{Hf{O}_{2}}$ in the first term is the electron affinity, band gap and the valence band maximum (VBM) of HfO_2_, respectively. As is well-known, DFT underestimates the oxide gap. And our calculation of HfO_2_ is 3.70 eV which is close to the result of 3.95eV^44,45^, indicating that our calculations are reliable. As shown in Fig. 2(a), the *p*-type Schottky-barrier height (SBH) ${\phi }_{p}$ is the difference between Ni Fermi energy (${E}_{F}^{Ni}$) and the ${E}_{VBM}^{Hf{O}_{2}}$, and both of them are given relative to the respective average of electrostatic potential, as obtained from two independent bulk calculations. Δ*V* is the difference between the macroscopic average potentials residing in Ni and HfO_2_ bulk-like-regions respectively^46^. The first term for equation (2) represents intrinsic bulk electronic structure of Ni and HfO_2_ bulk and is not related to the interface structure. While the second term Δ*V* is determined by the charge transfer that takes place in interfacial region. More detailed estimation of interface EWF can be found in our previous study^43^. For Ni(111)/Gr/HfO_2_(111) interface, when the graphene is inserted into Ni(111)/HfO_2_(111) interface, it would only change interfacial structure but Ni and HfO_2_ bulk electronic structures were not affected. Thus, according to equation (2), the change of EWF $\Delta {\varphi }_{eff}\,\,$would just stem from the change of Δ*V* that determined by the interface structure. Thus the underestimation of the band gap by the DFT does not affect the variation behavior of interface EWF induced by graphene intercalation and interfacial atom vacancy. Figure 2 shows the plane-averaged potentials for Ni(111)/HfO_2_(111) interface and Ni(111)/Gr/HfO_2_(111) interface with Ni combining onto Gr/HfO_2_ in top-hcp configuration. It can be evidently seen from Fig. 2 that, a substantial reduction of electrostatic potential exists in graphene layer, and the intercalation of graphene gives rise to a large increase of Δ*V* about 0.7 eV. That is, the EWF fells by about 0.7 eV. Both calculated results of Δ*V* for Ni(111)/Gr/HfO_2_(111) interfaces with Ni combining on Gr/HfO_2_ in the top-fcc and top-hcp configurations are the same. Furthermore, the calculated EWF of Ni(111)/Gr/HfO_2_(111) interface is about 5.3 eV being close to the PMOS’ requirement, which deviates the calculated work function value and other theoretical value^46,47^ of graphene (~4.5 eV), and is close to the value of Ni (~5.0 eV). This is very similar to the previous experimental report for Ni/graphene/SiO_2_^16^.

### Interfacial charge distribution and density of states for Ni(111)/Gr/HfO_2_(111) interface

The change of Δ*V* reflects the change of interface dipole barrier. And the change of interface dipole barrier originates in interfacial charge redistribution. To see the charge transfer effect, the plane averaged electron density difference of the valence electron ${\rm{\Delta }}\lambda (z)$ was considered, and it is defined as^47^

3 $${\rm{\Delta }}\lambda (z)=\frac{1}{A}\int [{n}_{A/B}(x,y,z)-{n}_{A}(x,y,z)-{n}_{B}(x,y,z)]dx\,dy.$$

**Figure 3:**
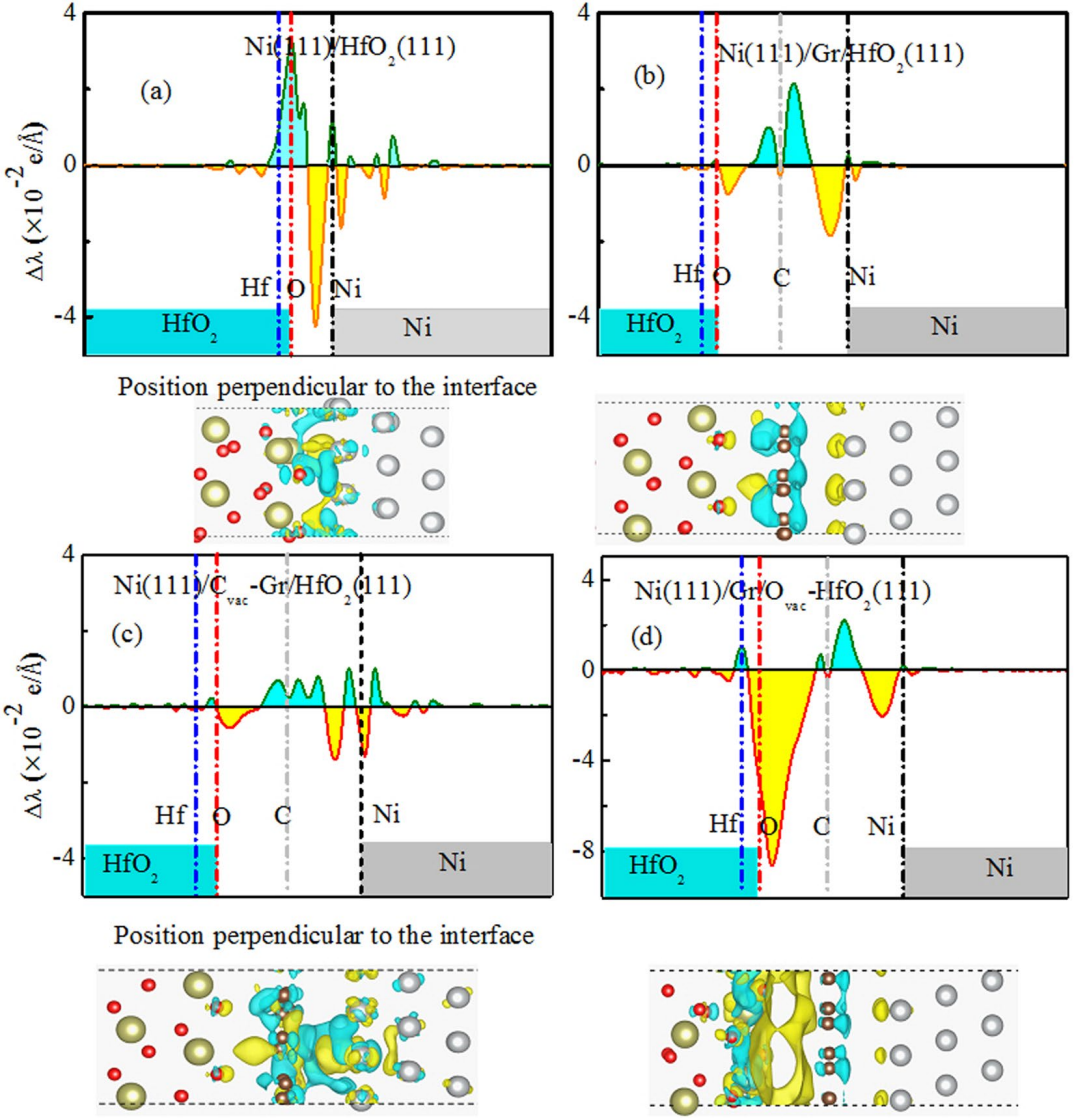
Plane averaged electron density difference of valence electron $\Delta \lambda (z)$ for (**a**) Ni(111)/HfO_2_(111), (**b**) Ni(111)/Gr/HfO_2_(111), (**c**) Ni(111)/C_vac_-Gr/HfO_2_(111), and (**d**) Ni(111)/Gr/O_vac_-HfO_2_(111) interfaces.

Here, z denotes the direction which is parallel to interface normal. ${n}_{A/B}(x,y,z)$, ${n}_{A}(x,y,z)$ and ${n}_{B}(x,y,z)$ are the electron density distributions of the interface and the component parts, respectively. Figure 3(a) and (b) show the plane averaged electron density difference of valence electron ${\rm{\Delta }}\lambda (z)$ for Ni(111)/HfO_2_(111) and Ni(111)/Gr/HfO_2_(111) interfaces, respectively. Blue and yellow denote electron accumulation and electron depletion, respectively. As shown in Fig. 3(a), there exists ionic bonding between interfacial O and Ni atoms for Ni(111)/HfO_2_(111) interface. The interface dipole is formed because of the electron accumulation near O layer and the electron depletion near interfacial Ni layer. And the electronic perturbations associated with the formation of interface extend into HfO_2_ and Ni side deeply. However, for Ni(111)/Gr/HfO_2_(111) interface, as shown in Fig. 3(b), the dipole layer does basically not move into HfO_2_ side, also just goes slightly deep into Ni side. The total electron transfer is obviously diminished compared to that for Ni(111)/HfO_2_(111) interface. This might be attributed to graphene’s unique aspects of charge screening by its relativistic low energy carriers, which can be clearly seen in Fig. 3(b). The behavior of interface dipoles is different from the one for Ni(111)/HfO_2_(111) interface. There are electron accumulation near graphene layer and electron depletion near interfacial O and Ni layers. Consequently, the total interface dipole comprises two parts: one is pointing from graphene layer to O layer and the other is pointing from graphene layer to Ni layer. Both parts are opposite and may be counteracted. Consequently, the whole dipole decreases. On the whole, the reduction of charge-transfer along with mutual competition between the two parts of interface dipole has led to the EWF to decrease.

**Figure 4:**
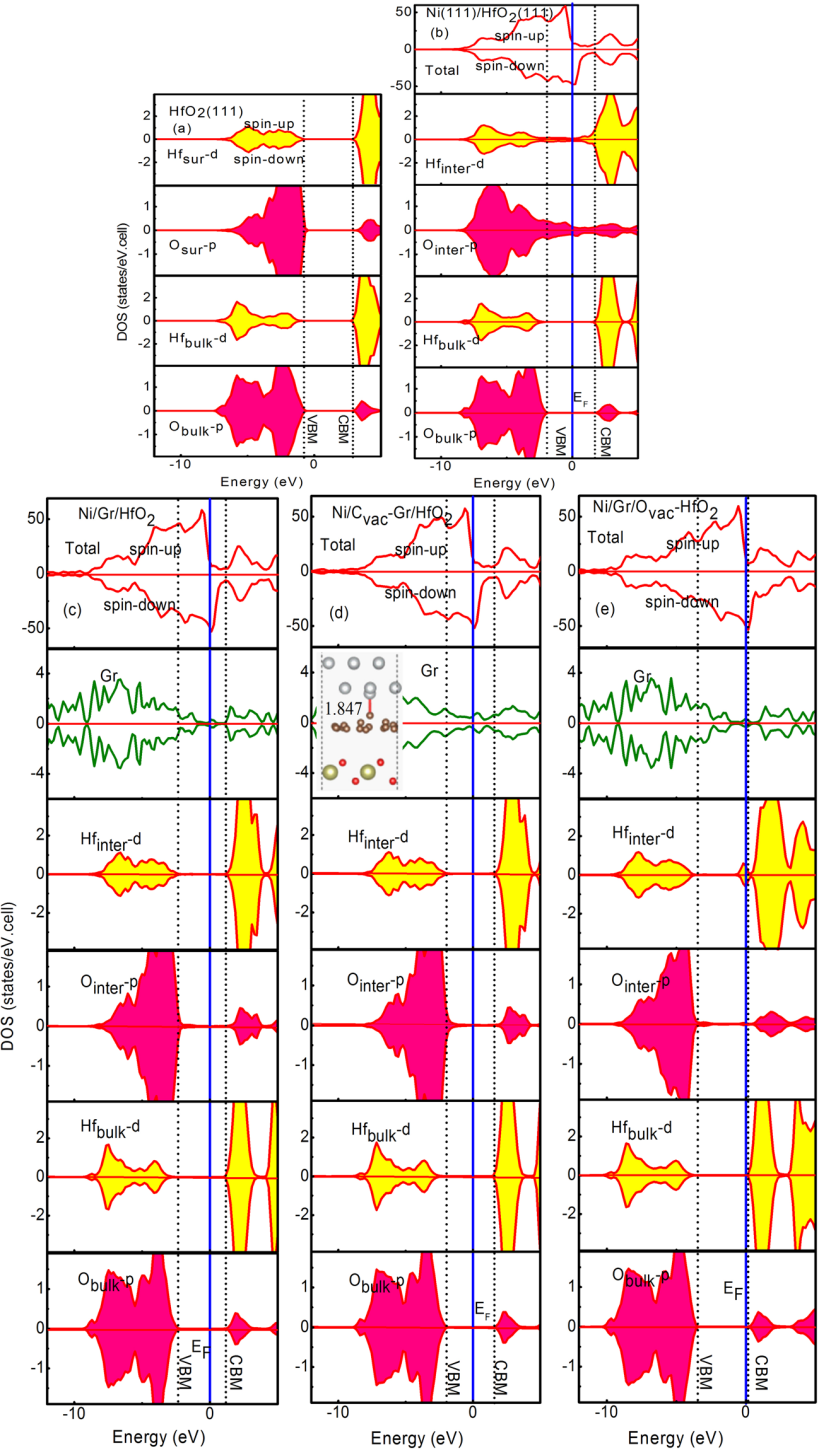
Total density of states (tDOS) and orbital-resolved partial density of states (PDOS) for (**a**) HfO_2_(111), (**b**) Ni(111)/HfO_2_(111), (**c**) Ni(111)/Gr/HfO_2_(111), (**d**) Ni(111)/C_vac_-Gr/HfO_2_(111), (**e**) Ni(111)/Gr/O_vac_-HfO_2_(111) interfaces. In the PDOS, the symbols are defined as follows: O and Hf in bulk region (O_bulk_, Hf_bulk_), interface O and Hf (O_inter_, Hf_inter_). The solid, dash, dot and dash lines denote Fermi energy of interface, VBM and CBM of HfO_2_, respectively.

Substantially, by controlling the relative position of Fermi level (*E*_F_), the EWF can be modulated. To better observe the variation behavior of Fermi level induced by graphene intercalating into Ni/HfO_2_ interface, we have plotted the density of states (DOS) for Ni(111)/HfO_2_(111) interfaces without and with graphene intercalation. Figure 4(b) and (c) show the total density of states (tDOS) and the orbital-resolved partial density of states (PDOS) of atoms residing in different layers for Ni(111)/HfO_2_(111) and Ni(111)/Gr/HfO_2_(111), respectively. Graphene intercalation into Ni/HfO_2_ interface shifts the Fermi level towards conduction band minimum (CBM) of HfO_2_. As a result, graphene intercalation acts as a donor dopant (n-type doping). Thus, it gives a well qualitative explanation for the EWF’s variation behavior. Moreover, as shown in Fig. 4(a), the PDOS of surfacial Hf and O atoms are very close to that of the inner ones for HfO_2_(111). However, comparing the PDOS of interfacial Hf and O atoms in Fig. 4(b) for Ni/HfO_2_ with that of surfacial Hf and O atoms for HfO_2_ in Fig. 4(a), it can clearly be found that the PDOS of interfacial Hf and O atoms differ very much from the surfacial ones for HfO_2_ without graphene intercalation. Especially, interfacial Hf and O atoms lost their dielectric properties but exhibited metallic properties. Thus interfacial interactions between Ni and HfO_2_ have induced contamination in HfO_2_ gate dielectrics and then degrade the dielectric reliability. However, for Ni(111)/Gr/HfO_2_(111) interface shown in Fig. 4(c), conversely, the PDOS of interfacial Hf and O atoms varies very little relative to that of inner ones. In other words, even for Hf and O atoms located at the boundary of the interface, they still remain their dielectric properties well. Thus, the dielectric properties of HfO_2_ gate dielectrics can be greatly improved by inserting a graphene layer between Ni and HfO_2_. This agrees with the experimental results reported by Misra *et al*.^18^ that inserting graphene between SiO_2_ and TiN has significantly improved dielectric reliability. We think that this improvement brought by graphene intercalation may be due to graphene’s excellent charge screening role on both Ni and HfO_2_ sides.

### Interfacial charge distribution and density of states for Ni(111)/Gr/HfO_2_(111) with interfacial atom vacancy

High dielectric reliability of oxide and effective work function tuning of metal-oxide interface are two important factors in determining the CMOS’s overall performance. In fact, experimentally, interfacial atom vacancy can be easy to form in interfacial layer for metal-oxide interface, and these defects should affect many properties of interface. In order to research the impacts of the interfacial atom vacancy, we study Ni/Gr/HfO_2_ interfaces with interfacial intrinsic atom vacancies in the interfacial layer: a small amount C vacancy with vacancy content of 1/18 monolayer (ML) in graphene layer, and O vacancy with a higher vacancy content of 1/4 ML in O atom layer closest to graphene layer, called Ni(111)/C_vac_-Gr/HfO_2_(111) and Ni(111)/Gr/O_vac_-HfO_2_(111) interfaces, respectively. The calculated DOS results for Ni(111)/C_vac_-Gr/HfO_2_(111) shown in Fig. 4(d) further demonstrate that even if there exists a small amount C vacancy in graphene layer, the Hf and O atoms located in interfacial region can also remain their original dielectric properties better. Morover, compared to the case for Ni(111)/Gr/HfO_2_(111) without C vacancy, graphene exhibits metallic properties. This may be attributed to chemical bonding between C and Ni atoms near C atomic vacancy. The bond length is about 1.847 Å which is shown in illustrations of Fig. 4(d). Moreover, we also assessed the EWF of Ni(111)/C_vac_-Gr/HfO_2_(111) interface, and it is found that C vacancy brought about an ascent of 0.3 eV compared to non-defective Ni(111)/Gr/HfO_2_(111). The calculated result of ΔV for Ni(111)/C_vac_-Gr/HfO_2_(111) is about 1.7 eV. According to Fig. 4(e), the relative movement trends for fermi energy may be a good explanation of the EWF’s ascent. Relative to the case of Ni(111)/Gr/HfO_2_(111), C vacancy acts as an acceptor dopant (p-type doping). This decline of EWF might be due to the change of charge transfer derived from C vacancy. Figure 3(c) shows the plane averaged electron density difference $\Delta \lambda (z)$ for Ni(111)/C_vac_-Gr/HfO_2_(111) interface. It can be clearly seen that electron transfer decreases compared with that for Ni(111)/Gr/HfO_2_(111) shown in Fig. 3(b).

As seen in Fig. 4(e), for Ni(111)/Gr/O_vac_-HfO_2_(111), it can be easy to find that interfacial Hf and O atoms also remain their dielectric properties. Interfacial O vacancy does hardly change graphene’s properties. It is worthwhile noted that, compared with the case of non-defective Ni(111)/Gr/HfO_2_(111), interfacial O vacancies only bring about an EWF’s decline of 1.0 eV. The calculated result of ΔV for Ni(111)/Gr/O_vac_-HfO_2_(111) is about 3.0 eV. In other words, interfacial O vacancy can effectively tune the EWF from 5.3 eV for non-defective Ni/Gr/HfO_2_ to 4.3 eV (being close to the NMOS’ requirement) without sacrificing dielectric reliability of gate dielectric, which is fascinating and desirable. The EWF’s big reduction is associated with interfacial charge redistribution. Seen in Fig. 3(d), a large amount of electron depletion appear near interfacial O layer. The EWF’s reduction can also be ascribed to the defect states induced by interfacial O vacancies which can be seen clearly in PDOS of Hf_inter_-d in Fig. 4(e). This is similar to the result that oxygen vacancy has n-type doping effect for Gr/HfO_2_, obtained by Chiu *et al*.^36^. Corresponding to the relative movement behavior for the fermi energy, contrary to the case for C vacancy, interfacial O vacancy acts as a donor dopant (n-type doping).

In summary, we have investigated the effects of graphene intercalation on dielectric reliability of HfO_2_, and the effects of graphene intercalation and interfacial oxygen or carbon vacancy on the effective work function of Ni/Gr/HfO_2_ interface using first-principle calculation. It is found that the graphene intercalation reduces metallic contamination in HfO_2_ dielectric even for those interfaces with interfacial O vacancy and a small amount C vacancy. Consequently, the graphene intercalation improves the dielectric reliability of HfO_2_ and the interface quality. Our work strongly indicates that the use of graphene synthesized into Ni/HfO_2_ interface can significantly improve dielectric reliability of gate dielectric and then to effectively reduce the physical thickness of gate dielectric. In addition, controlling interfacial oxygen or carbon vacancy is a very effective way for modulating the EWF of Ni/Gr/HfO_2_ interface. By inserting graphene into Ni/HfO_2_ interface, the EWF could drop about 0.7 eV. Furthermore, only the removal of 1/18 ML carbon atom in graphene layer could induce a considerable EWF increment of 0.3 eV, while the removal of 1/4 ML interfacial oxygen atom could give rise to a great EWF decrement of 1.0 eV. These are very important for metal gate technology and application where the EWF of metal/oxide interface needs to be modulated. And these results also have practical significance for the realization of high-performance graphene-based devices.
